# The Secret of Centenarianism

**Published:** 1896-03

**Authors:** 


### The Secret of Centenarianism.

A "chiel" from *Tit-Bits* has been with Sir Benjamin Ward Richardson "takin' notes" of his opinions on things in general, which he has printed for the edification of the readers of that educational periodical. The eminent physician fought all his scientific battles o'er again, and confided to his appreciative listener many interesting details as to his professional career. With these we have no concern at present. On one point, however, as to which the interviewer was particularly eager to hear the deliverance of the oracle, the reader will doubtless to some extent share his curiosity. Sir Benjamin gave it as his "fixed opinion that every man, and every woman for that matter, should attain the age of one hundred." He proceeded to show how this was to be done. First of all, as we gather, the would-be centenarian must have "light hazel eyes, light brown hair, complexion inclined to be florid, lips and eyelids of a good natural red—never pale, and rarely of a bluish tint." Then he must never smoke and never drink—the prohibition is absolute, but we presume the restriction applies only to alcoholic liquors; further, he should eat very little meat. He should work as little as possible by artificial light; in fact, one of Sir Benjamin's most widely quoted sayings, we are told, is: "Make the sun your fellow workman." If, by the way, this rule is strictly adhered to in this country, few people are likely to die of overwork. What the color of the eye may have to do with longevity does not seem to have been revealed to the interviewer. An American authority professes to be able to diagnose a predisposition to centenarianism by the length and breadth of the head; he says nothing as to its thickness, which yet may help to make a man's days long in the land. As to the rigid abstinence from tobacco and alcohol enjoined by Sir Benjamin Ward Richardson on all candidates for the long-distance race of life, it has almost as slight a basis of fact as the importance he attaches to the color of the eyes. Immoderate drinking of whisky, like immoderate drinking of tea, or for that matter immoderate eating of bread, will shorten life; but what evidence is available on the subject seems to show that a strictly temperate use of alcohol tends to prolong life, for the excellent reason that it assists digestion and thereby promotes health. The most trustworthy statistics on this subject are those of Sir George Humphry. Of forty-five cases of centenarians collected by him only twelve were total abstainers, while thirty were moderate drinkers, and three were heavy drinkers. Of 689 persons between eighty and one hundred years of age in Sir George Humphry's tables only a fraction over 12 percent were abstainers, while nearly 9 percent were heavy drinkers. The abstainers would appear from these figures to have only a slight advantage in point of longevity over the non-abstainers. The real secret of centenarianism is well expressed by Sir George Humphry when he says: "The prime requisite is the faculty of age in the blood by inheritance." In other words, if you wish to live a hundred years you must, as Oliver Wendell Holmes said of another matter, begin by going back two or three hundred years, and securing for yourself a sound and long-lived ancestry.—*British Med. Journal*

The above selection contains some thoughts that are worthy of consideration. The subject of general hygiene that underlies the suggestion, is one with which all ought to be more familiar—is one to which, in a practical way, the attention of the medical profession should be more specially given, from the highest medical college in the country, all the way to the most modest, unassuming country practitioner.

In the above are some rather peculiar statements, for instance: "The would-be centenarian must have light hazel eyes; light brown hair; complexion inclined to be florid; lips and eyelids of of a good natural red, never pale, and rarely of a bluish tint."

These conditions are of no special importance, except so far as their presence may indicate a good texture of the organs and tissues of the body so well wrought up and woven together that they will be able to maintain their integrity for a relatively great period of time.

The complexion inclined to be florid, lips and eye lids of a good natural red indicate excellent tissue of the skin of the analogous structures as well as a good circulation. In order that the well-organized body may attain the greatest age, it is important that it shall have those conditions and supplies that are in the best degree tributary to its maintenance and that it shall be free from all those things and conditions that would, in any degree, be injurious to it. In this article some habits are referred to and apologized for, that certainly ought not to be encouraged; certain statistics are presented to show that abstinence from tobacco and alcohol does not promote longevity, and even the statistics of Sir Geo. Humphry, here referred to, fail to prove their non-injurious character. Every effort hitherto made to demonstrate that the use of tobacco and alcohol are beneficial to the animal economy have been significant failures, while the observation and too frequent experience of multitudes of people is that the use of these two articles have wrought more injury to the human race than the use of any other two materials with which we have to do—have wrought more ruin and misery than war, pestilence and famine.

It is a surprise that a journal, with the broad and full knowledge of everything that pertains to our physical well-being, should permit such an apology for two great evils to appear on its pages. —[Ed. Register.]

